# Supplementary material for: Transcontinental dispersal of Anopheles gambiae occurred from West African origin via serial founder events
Source: Commun Biol. 2019 Dec 19;2:473. doi: 10.1038/s42003-019-0717-7 (PMC6923408; doi:10.1038/s42003-019-0717-7)
Supplement: Supplementary file 1 — Supplementary Information [file 42003_2019_717_MOESM1_ESM.pdf]

Supplementary Information

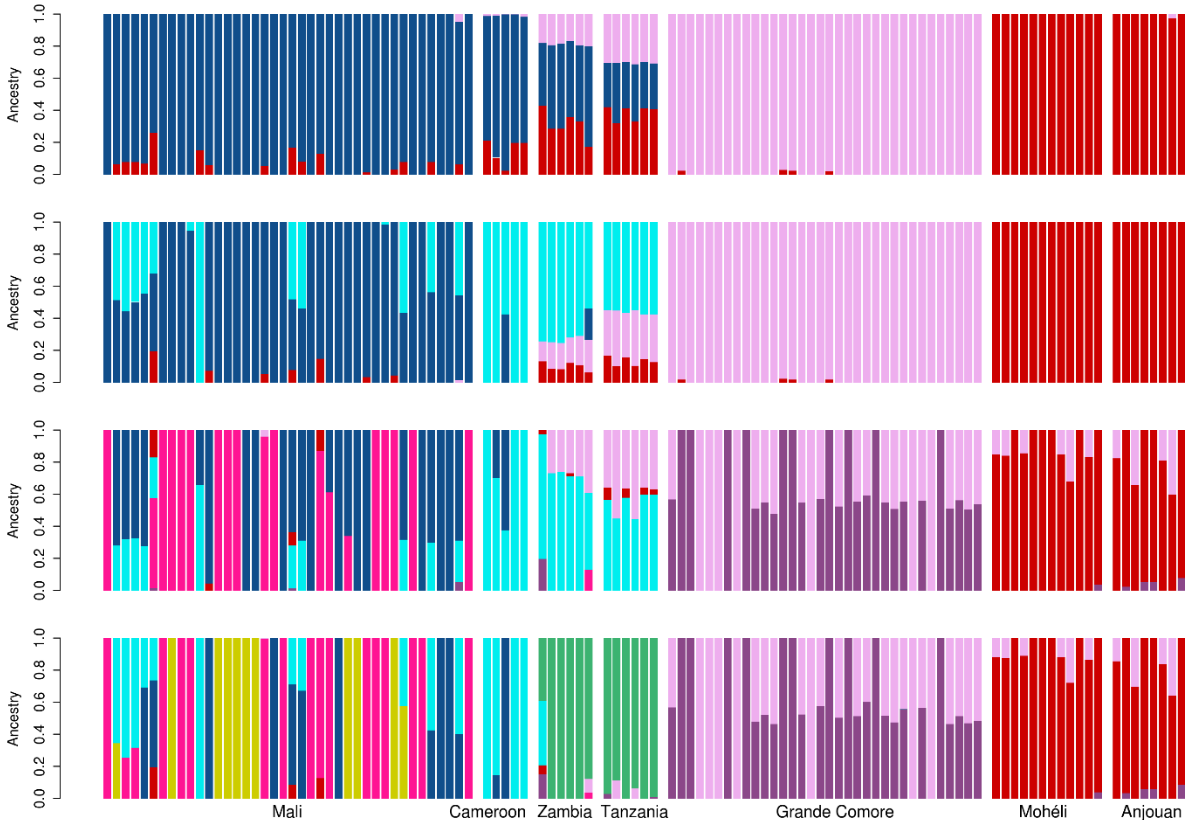

Supplementary Figure 1 – ADMIXTURE analysis

Shown are the results for K=3, K=4, K=6 and K=8 (from top to bottom).

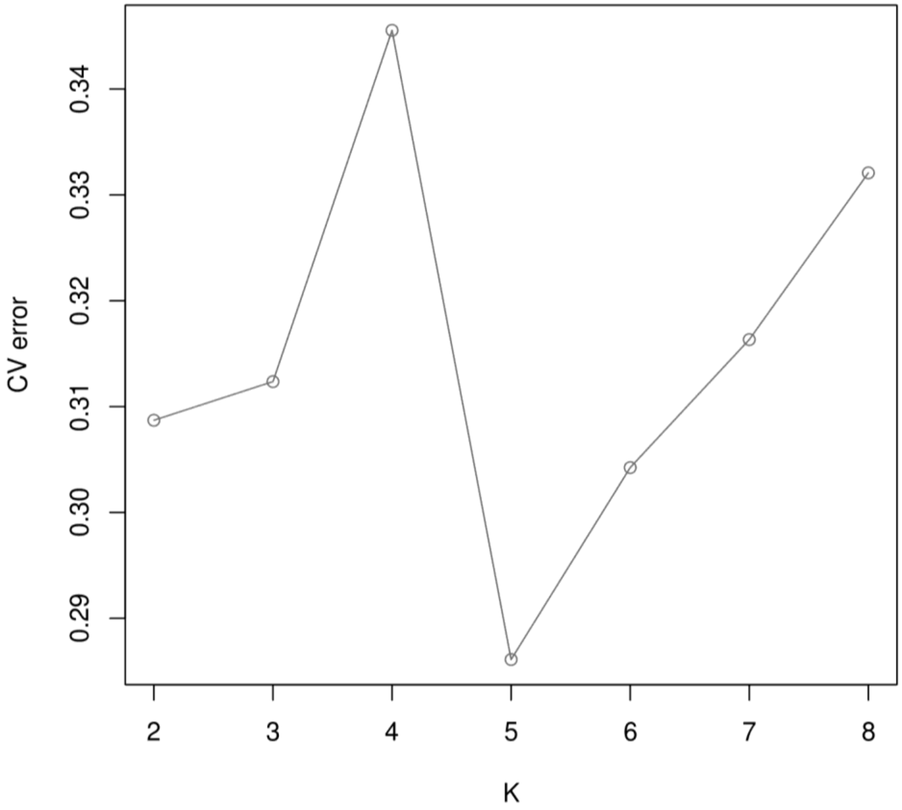

Supplementary Figure 2 – CV error estimates for ADMIXTURE analyses with different K's

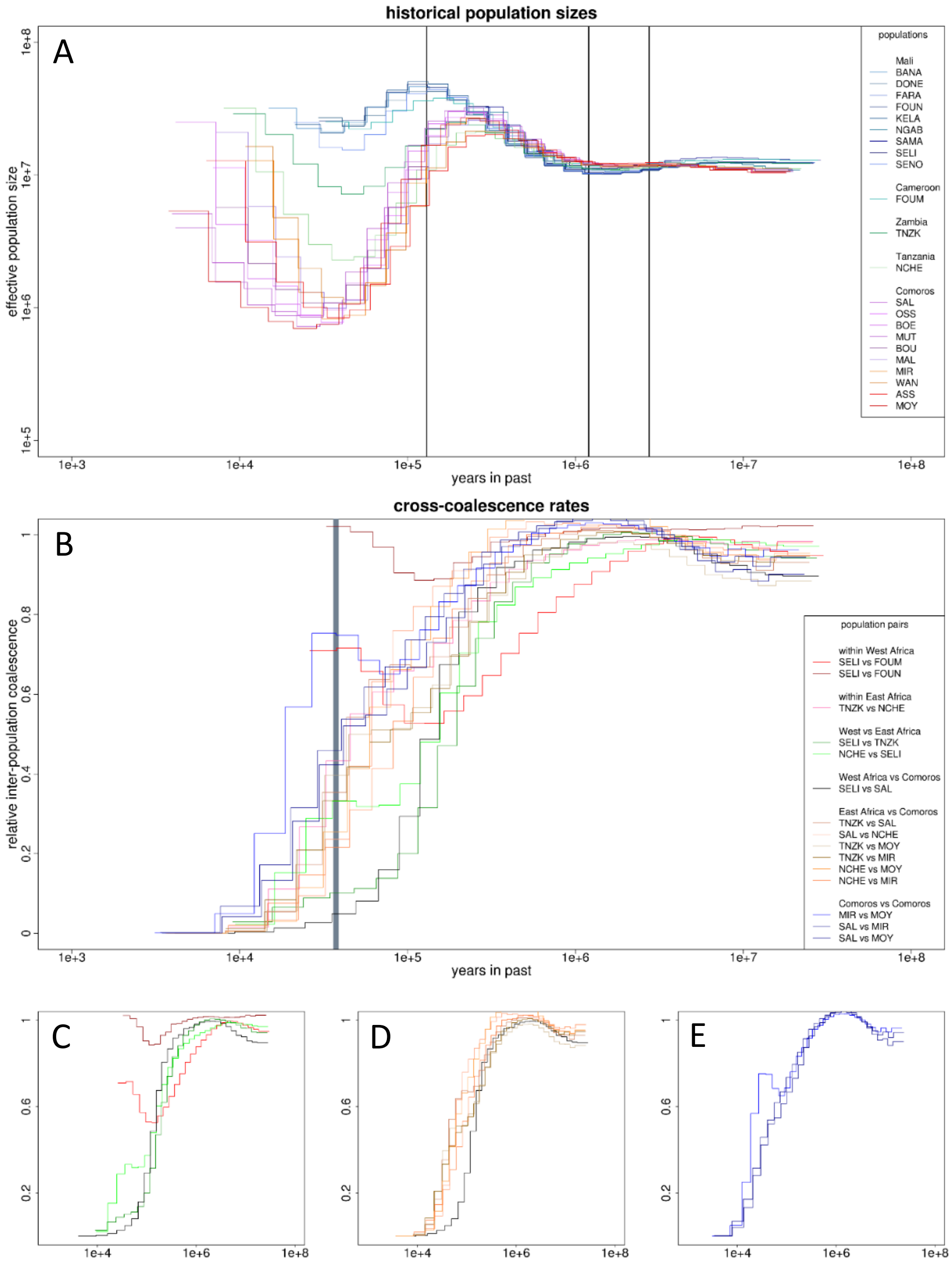

Supplementary Figure 3 – MSMC2 results

(A) Historical effective population sizes per site. The vertical lines depict the geological appearance of the three islands of the Comoros sampled. (B-E) Cross-coalescence rates of inter-population comparisons. (B) The vertical bar marks the time point most closely to present where estimates for all comparisons are available. (C) Subset of B with only population comparisons of Mali vs other sites. (D) Subset of B with only Comoro vs mainland populations. (E) Subset of B with only inter-Comoro comparisons. Color schemes in C-E are the same as in B. Note the logarithmic scales in plots (all but y-axes in B-E).

Supplementary Table 1 - Statistics per sample

Samples can be found in NCBI GenBank under BioProject ID PRJNA590708.

sample	NCBI accession number	# raw reads	% mapped reads	Medium insert size	coverage
06SAMA0103	SAMN13337315	22,045,130	97.78%	429	8.0
06SAMA0130	SAMN13337316	23,756,245	98.07%	427	8.6
06FOUN0009	SAMN13337317	42,565,747	94.19%	407	14.8
06FOUN0010	SAMN13337318	56,097,824	90.67%	368	18.8
06FOUN0026	SAMN13337319	25,316,623	90.22%	405	8.5
06FOUN0028	SAMN13337320	20,774,239	93.10%	400	7.2
06DONE0022	SAMN13337321	22,844,443	95.41%	401	8.1
06DONE0044	SAMN13337322	52,238,457	89.33%	393	17.3
06DONE0045	SAMN13337323	50,681,550	92.93%	380	17.4
06DONE0046	SAMN13337324	23,051,498	85.91%	404	7.3
06DONE0104	SAMN13337325	42,555,805	93.45%	421	14.7
06DONE0132	SAMN13337326	20,050,934	95.48%	422	7.1
06BANA0008	SAMN13337327	39,531,310	90.14%	400	13.2
06BANA0010	SAMN13337328	30,501,977	93.57%	333	10.6
06BANA0012	SAMN13337329	29,949,432	89.43%	389	9.9
06BANA0013	SAMN13337330	50,438,159	94.72%	351	17.7
06BANA0016	SAMN13337331	22,003,804	92.56%	424	7.5
06BANA0035	SAMN13337332	20,886,896	95.82%	364	7.4
2006NGAB901E	SAMN13337333	54,121,189	98.64%	389	19.8
06SENO0003	SAMN13337334	22,767,308	91.54%	421	7.7
06SENO0009	SAMN13337335	20,707,512	98.46%	401	7.6
06SENO0021	SAMN13337336	19,005,670	98.17%	420	6.9
06SENO0024	SAMN13337337	49,386,568	98.05%	413	17.9
06SENO0031	SAMN13337338	22,797,847	87.57%	412	7.4
06SENO0035	SAMN13337339	21,437,580	89.65%	427	7.1
06FARA0003	SAMN13337340	30,276,335	91.94%	400	10.3
06FARA0004	SAMN13337341	49,210,477	91.05%	399	16.6
06FARA0007	SAMN13337342	23,441,093	94.48%	415	8.2
06FARA0008	SAMN13337343	27,015,461	97.67%	399	9.8
06FARA0009	SAMN13337344	22,953,867	98.10%	417	8.3
06KELA0003	SAMN13337345	22,044,504	98.75%	405	8.1
06KELA0016	SAMN13337346	52,310,238	98.44%	411	19.1
06KELA0066	SAMN13337347	20,346,700	97.97%	405	7.4
06KELA0084	SAMN13337348	19,868,669	98.51%	405	7.2
06KELA0106	SAMN13337349	17,095,249	98.63%	421	6.2
06SELI0102	SAMN13337350	20,456,085	96.27%	441	7.3
06SELI0106	SAMN13337351	58,579,044	92.80%	305	20.1
06SELI0114	SAMN13337352	23,034,202	93.58%	399	8.0
06SELI0117	SAMN13337353	20,726,953	96.36%	429	7.4
06SELI0123	SAMN13337354	64,363,548	96.87%	403	23.1
06FOUM0032	SAMN13337355	22,249,164	95.54%	419	7.9
06FOUM0033	SAMN13337356	22,534,380	90.74%	420	7.6

sample	NCBI accession number	# raw reads	% mapped reads	Medium insert size	coverage
06FOUM0035	SAMN13337357	91,730,833	96.17%	361	32.7
06FOUM0036	SAMN13337358	32,191,373	98.32%	417	11.7
06FOUM0037	SAMN13337359	20,476,425	94.46%	392	7.2
2012TNZK004	SAMN13337360	30,482,811	97.91%	350	11.1
2012TNZK039	SAMN13337361	58,800,000	98.59%	324	21.5
2012TNZK042	SAMN13337362	26,662,011	98.27%	347	9.7
2012TNZK044	SAMN13337363	34,466,136	97.05%	367	12.4
2012TNZK048	SAMN13337364	31,895,511	98.37%	321	11.6
2012TNZK057	SAMN13337365	28,687,884	98.01%	327	10.4
2015NCHE001	SAMN13337366	26,854,122	96.29%	404	9.6
2015NCHE002	SAMN13337367	23,339,226	98.59%	438	8.5
2015NCHE003	SAMN13337368	28,837,835	86.61%	366	9.3
2015NCHE004	SAMN13337369	31,765,127	90.28%	380	10.6
2015NCHE005	SAMN13337370	44,781,499	98.51%	364	16.3
2015NCHE006	SAMN13337371	27,283,888	97.63%	412	9.9
2011BOU003	SAMN13337372	27,677,011	96.89%	386	9.9
2011BOU015	SAMN13337373	24,672,148	98.09%	390	9.0
2011BOU019	SAMN13337374	23,117,304	96.83%	403	8.3
2011BOU029	SAMN13337375	20,383,883	98.54%	400	7.4
2011BOU030	SAMN13337376	23,814,955	97.62%	393	8.6
2011BOU042	SAMN13337377	50,555,233	96.45%	398	18.1
2011BOE005	SAMN13337378	23,597,208	97.66%	400	8.5
2011BOE007	SAMN13337379	23,948,978	98.24%	407	8.7
2011BOE020	SAMN13337380	23,726,039	98.43%	410	8.6
2011BOE035	SAMN13337381	60,884,880	97.67%	260	22.0
2011BOE036	SAMN13337382	25,832,367	97.82%	250	9.4
2011BOE048	SAMN13337383	29,477,875	97.55%	395	10.7
2011OSS001	SAMN13337384	18,415,486	98.69%	418	6.7
2011OSS012	SAMN13337385	22,492,203	98.67%	408	8.2
2011OSS013	SAMN13337386	20,531,472	97.82%	421	7.4
2011OSS017	SAMN13337387	20,844,334	98.32%	415	7.6
2011OSS018	SAMN13337388	49,813,232	98.66%	395	18.2
2011OSS027	SAMN13337389	23,657,169	98.51%	411	8.6
2011MUT002	SAMN13337390	24,123,640	98.78%	375	8.8
2011MUT038	SAMN13337391	26,549,241	91.82%	418	9.0
2011MUT039	SAMN13337392	61,654,286	90.30%	349	20.6
2011MUT041	SAMN13337393	42,833,005	96.08%	415	15.2
2011MUT045	SAMN13337394	22,795,876	95.25%	430	8.0
2011MUT046	SAMN13337395	21,616,511	93.74%	421	7.5
2011SAL001	SAMN13337396	20,818,098	98.51%	432	7.6
2011SAL003	SAMN13337397	23,421,787	97.97%	440	8.5
2011SAL005	SAMN13337398	59,656,534	98.28%	400	21.7
2011SAL008	SAMN13337399	19,583,349	98.80%	413	7.2
2011MAL006	SAMN13337400	45,050,379	97.90%	296	16.3
2011MAL017	SAMN13337401	69,276,716	98.74%	297	25.3
2011MAL020	SAMN13337402	26,061,794	98.12%	314	9.5
2011MAL028	SAMN13337403	22,911,609	98.37%	355	8.3
2011MAL083	SAMN13337404	34,118,415	97.31%	288	12.3

sample	NCBI accession number	# raw reads	% mapped reads	Medium insert size	coverage
2011MAL088	SAMN13337405	29,032,425	97.65%	322	10.5
2011MIR019	SAMN13337406	25,303,206	97.75%	386	9.2
2011MIR032	SAMN13337407	28,069,375	97.56%	393	10.1
2011MIR033	SAMN13337408	27,654,576	97.24%	374	10.0
2011MIR036	SAMN13337409	91,169,806	96.13%	341	32.5
2011MIR056	SAMN13337410	25,092,357	96.53%	382	9.0
2011MIR058	SAMN13337411	25,570,335	96.78%	377	9.2
2011WAN033	SAMN13337412	33,242,529	97.50%	297	12.0
2011WAN035	SAMN13337413	28,550,801	97.23%	307	10.3
2011WAN041	SAMN13337414	31,590,508	96.12%	342	11.2
2011WAN042	SAMN13337415	83,751,592	96.81%	316	30.0
2011WAN043	SAMN13337416	90,390,954	96.85%	316	32.4
2011WAN045	SAMN13337417	27,397,435	96.55%	352	9.8
2011ASS097	SAMN13337418	24,931,971	98.58%	373	9.1
2011ASS195	SAMN13337419	25,499,747	97.06%	398	9.2
2011ASS213	SAMN13337420	20,935,598	98.21%	393	7.6
2011MOY057	SAMN13337421	51,606,695	92.20%	403	17.6
2011MOY063	SAMN13337422	26,306,902	91.74%	410	8.9
2011MOY069	SAMN13337423	24,675,282	88.39%	430	8.1
2011MOY072	SAMN13337424	24,530,149	92.45%	396	8.4
2011MOY073	SAMN13337425	36,126,723	75.67%	399	10.1
	**MEAN**	33,113,661	0.96%	385	11.7
	**MEDIAN**	26,061,794	0.97%	399	9.2
	**MIN**	17,095,249	0.76%	250	6.2
	**MAX**	91,730,833	0.99%	441	32.7

Supplementary Table 2 – F_{ST} values for all inter-population comparisons.

Standard deviation provided in parenthesis.

Pop	BANA	DONE	FARA	FOUN	KELA	SAMA	SEL	SENO	FOUM	ASS	BOE	BOU	MAL	MIR	MOY	MUT	OSS	SAL	WAN	TNZK	NCHE
**BANA**																					
**DONE**	-0.0030 (0.0002)																				
**FARA**	0.0094 (0.0003)	-0.0016 (0.0002)																			
**FOUN**	-0.0041 (0.0002)	-0.0048 (0.0002)	0.0055 (0.0003)																		
**KELA**	-0.0039 (0.0002)	-0.0026 (0.0002)	0.0068 (0.0002)	-0.0106 (0.0002)																	
**SAMA**	-0.0131 (0.0004)	-0.0210 (0.0003)	-0.0290 (0.0003)	-0.0186 (0.0003)	-0.0158 (0.0003)																
**SEL**	-0.0044 (0.0002)	-0.0071 (0.0002)	-0.0008 (0.0002)	-0.0088 (0.0003)	-0.0080 (0.0002)	-0.0193 (0.0003)															
**SENO**	-0.0019 (0.0002)	-0.0020 (0.0002)	0.0059 (0.0002)	-0.0077 (0.0002)	-0.0067 (0.0002)	-0.0159 (0.0003)	-0.0079 (0.0002)														
**FOUM**	0.0180 (0.0003)	0.0293 (0.0005)	0.0422 (0.0007)	0.0310 (0.0006)	0.0321 (0.0006)	0.0231 (0.0008)	0.0229 (0.0005)	0.0309 (0.0005)													
**ASS**	0.1785 (0.0011)	0.1932 (0.0011)	0.2035 (0.0011)	0.1849 (0.0011)	0.1959 (0.0012)	0.1766 (0.0011)	0.1871 (0.0011)	0.1995 (0.0011)	0.1832 (0.0011)												
**BOE**	0.1539 (0.0008)	0.1610 (0.0008)	0.1671 (0.0008)	0.1581 (0.0009)	0.1654 (0.0009)	0.1405 (0.0008)	0.1541 (0.0008)	0.1636 (0.0008)	0.1599 (0.0008)	0.1982 (0.0016)											
**BOU**	0.1576 (0.0009)	0.1682 (0.0008)	0.1766 (0.0008)	0.1665 (0.0009)	0.1732 (0.0009)	0.1499 (0.0009)	0.1619 (0.0008)	0.1706 (0.0008)	0.1594 (0.0009)	0.1971 (0.0017)	0.0468 (0.0008)										
**MAL**	0.1455 (0.0008)	0.1575 (0.0008)	0.1677 (0.0009)	0.1561 (0.0008)	0.1626 (0.0009)	0.1416 (0.0009)	0.1514 (0.0008)	0.1606 (0.0008)	0.1443 (0.0008)	0.1817 (0.0016)	0.0427 (0.0008)	0.0403 (0.0008)									
**MIR**	0.1660 (0.0008)	0.1807 (0.0009)	0.1924 (0.0010)	0.1787 (0.0009)	0.1863 (0.0009)	0.1661 (0.0011)	0.1754 (0.0009)	0.1859 (0.0009)	0.1629 (0.0008)	0.1311 (0.0016)	0.1920 (0.0012)	0.1885 (0.0013)	0.1674 (0.0012)								
**MOY**	0.1812 (0.0010)	0.1963 (0.0010)	0.2075 (0.0010)	0.1905 (0.0010)	0.2005 (0.0011)	0.1807 (0.0011)	0.1905 (0.0010)	0.2023 (0.0010)	0.1815 (0.0010)	-0.0066 (0.0011)	0.1985 (0.0014)	0.1946 (0.0015)	0.1758 (0.0014)	0.1280 (0.0014)							
**MUT**	0.1521 (0.0009)	0.1639 (0.0008)	0.1738 (0.0009)	0.1626 (0.0009)	0.1692 (0.0009)	0.1473 (0.0010)	0.1582 (0.0008)	0.1670 (0.0008)	0.1516 (0.0008)	0.1869 (0.0017)	0.0295 (0.0007)	0.0323 (0.0008)	0.0134 (0.0007)	0.1754 (0.0013)	0.1835 (0.0015)						
**OSS**	0.1525 (0.0009)	0.1590 (0.0008)	0.1644 (0.0008)	0.1560 (0.0009)	0.1631 (0.0009)	0.1375 (0.0008)	0.1519 (0.0008)	0.1613 (0.0008)	0.1596 (0.0008)	0.1962 (0.0016)	0.0362 (0.0008)	0.0460 (0.0008)	0.0438 (0.0007)	0.1927 (0.0012)	0.1971 (0.0014)	0.0414 (0.0007)					
**SAL**	0.1628 (0.0009)	0.1717 (0.0009)	0.1788 (0.0008)	0.1694 (0.0009)	0.1763 (0.0010)	0.1516 (0.0009)	0.1651 (0.0009)	0.1746 (0.0009)	0.1666 (0.0009)	0.2054 (0.0018)	0.0649 (0.0011)	0.0567 (0.0011)	0.0367 (0.0009)	0.1952 (0.0013)	0.2030 (0.0016)	0.0452 (0.0010)	0.0524 (0.0010)				
**WAN**	0.1568 (0.0008)	0.1707 (0.0009)	0.1825 (0.0010)	0.1681 (0.0009)	0.1758 (0.0009)	0.1564 (0.0010)	0.1651 (0.0009)	0.1752 (0.0009)	0.1550 (0.0008)	0.1203 (0.0016)	0.1809 (0.0012)	0.1767 (0.0013)	0.1564 (0.0012)	0.0022 (0.0006)	0.1162 (0.0014)	0.1653 (0.0013)	0.1807 (0.0012)	0.1837 (0.0013)			
**TNZK**	0.0917 (0.0006)	0.0966 (0.0006)	0.1022 (0.0005)	0.0932 (0.0006)	0.0990 (0.0006)	0.0769 (0.0006)	0.0890 (0.0005)	0.0983 (0.0006)	0.1016 (0.0006)	0.1818 (0.0013)	0.1364 (0.0009)	0.1433 (0.0009)	0.1304 (0.0009)	0.1648 (0.0009)	0.1826 (0.0012)	0.1371 (0.0009)	0.1347 (0.0009)	0.1493 (0.0012)	0.1533 (0.0009)		
**NCHE**	0.0422 (0.0005)	0.0499 (0.0004)	0.0604 (0.0005)	0.0478 (0.0005)	0.0524 (0.0005)	0.0365 (0.0006)	0.0427 (0.0004)	0.0514 (0.0005)	0.0446 (0.0004)	0.1541 (0.0012)	0.1223 (0.0008)	0.1247 (0.0009)	0.1105 (0.0008)	0.1363 (0.0008)	0.1534 (0.0011)	0.1174 (0.0008)	0.1211 (0.0008)	0.1311 (0.0010)	0.1259 (0.0008)	0.0583 (0.0006)	

Supplementary Table 3 – Information population sampling

Country	Site	Abbreviation	Latitude	Longitude	Year	N
Mali	Banambani	BANA	12.8	-8.05	2006	6
Mali	Doneguebougou	DONE	12.80683	-7.98476	2006	6
Mali	Farada	FARA	12.43664	-8.20617	2006	5
Mali	Founia	FOUN	12.89163	-9.46063	2006	4
Mali	Kela	KELA	11.88683	-8.44744	2006	5
Mali	Ngabokoro Droit	NGAB	12.6935	-7.8499	2006	1
Mali	Samakorola	SAMA	13.9167	-8.0833	2006	2
Mali	Selinkenyi	SELI	11.7	-8.2833	2006	5
Mali	Senou	SENO	12.5167	-7.9167	2006	6
Cameroon	Foumbot	FOUM	5.4851	10.6	2006	5
Tanzania	Dar Es Salaam	6	-6.8333	39.2667	2012	6
Zambia	Nchelenge	NCHE	-9.3333	28.7167	2015	6
Comoros	Assimpao	ASS	-12.2373	44.3165	2011	3
Comoros	Boeninidi	BOE	-11.5659	43.2872	2011	6
Comoros	Bouni	BOU	-11.4894	43.3975	2011	6
Comoros	Malé	MAL	-11.8865	43.5063	2011	6
Comoros	Miringoni	MIR	-12.302	43.6372	2011	6
Comoros	Moya	MOY	-12.3093	44.4395	2011	5
Comoros	Mutsamudu	MUT	-11.6099	43.3903	2011	6
Comoros	Ossivo	OSS	-11.5884	43.2776	2011	6
Comoros	Saliman	SAL	-11.6803	43.2661	2011	4
Comoros	Wanani	WAN	-12.3451	43.8001	2011	6
