# Supplementary material for: Transcontinental dispersal of Anopheles gambiae occurred from West African origin via serial founder events
Source: Commun Biol. 2019 Dec 19;2:473. doi: 10.1038/s42003-019-0717-7 (PMC6923408; doi:10.1038/s42003-019-0717-7)
Supplement: Supplementary file 2 — Reporting Summary [file 42003_2019_717_MOESM2_ESM.pdf]

nature research

Corresponding author(s): Hanno Schmidt

Last updated by author(s): 2019-11-21

Reporting Summary

Nature Research wishes to improve the reproducibility of the work that we publish. This form provides structure for consistency and transparency in reporting. For further information on Nature Research policies, see Authors & Referees and the Editorial Policy Checklist.

Statistics

For all statistical analyses, confirm that the following items are present in the figure legend, table legend, main text, or Methods section.

n/a	Confirmed	
☐	☒	The exact sample size (*n*) for each experimental group/condition, given as a discrete number and unit of measurement
☐	☒	A statement on whether measurements were taken from distinct samples or whether the same sample was measured repeatedly
☐	☒	The statistical test(s) used AND whether they are one- or two-sided<br>*Only common tests should be described solely by name; describe more complex techniques in the Methods section.*
☐	☒	A description of all covariates tested
☐	☒	A description of any assumptions or corrections, such as tests of normality and adjustment for multiple comparisons
☐	☒	A full description of the statistical parameters including central tendency (e.g. means) or other basic estimates (e.g. regression coefficient) AND variation (e.g. standard deviation) or associated estimates of uncertainty (e.g. confidence intervals)
☐	☒	For null hypothesis testing, the test statistic (e.g. *F*, *t*, *r*) with confidence intervals, effect sizes, degrees of freedom and *P* value noted<br>*Give P values as exact values whenever suitable.*
☐	☒	For Bayesian analysis, information on the choice of priors and Markov chain Monte Carlo settings
☐	☒	For hierarchical and complex designs, identification of the appropriate level for tests and full reporting of outcomes
☐	☒	Estimates of effect sizes (e.g. Cohen's *d*, Pearson's *r*), indicating how they were calculated

Our web collection on statistics for biologists contains articles on many of the points above.

Software and code

Policy information about availability of computer code

Data collection	No software was used for data collection.
Data analysis	Trimmomatic v0.36, Picard Tools v2.2.4, GATK v3.5, BWA-MEM v0.7.15, Freebayes v1.0.1, R v3.2.5, SpaceMix v0.13, Raddle, VCFtools v0.1.12b , PHYLIP v3.696, ADMIXTURE v1.3.0, SHAPEIT2 v2.9, MSMC2 v2.0.2, BEDOPS v2.4.30

For manuscripts utilizing custom algorithms or software that are central to the research but not yet described in published literature, software must be made available to editors/reviewers. We strongly encourage code deposition in a community repository (e.g. GitHub). See the Nature Research guidelines for submitting code & software for further information.

Data

Policy information about availability of data

All manuscripts must include a data availability statement. This statement should provide the following information, where applicable:

- Accession codes, unique identifiers, or web links for publicly available datasets
- A list of figures that have associated raw data
- A description of any restrictions on data availability

Sequence data that support the findings of this study are deposited in NCBI GenBank with accession numbers SAMN13337315-SAMN13337425 under BioProject ID PRJNA590708.

Field-specific reporting

Please select the one below that is the best fit for your research. If you are not sure, read the appropriate sections before making your selection.

☐ Life sciences ☐ Behavioural & social sciences ☒ Ecological, evolutionary & environmental sciences

Ecological, evolutionary & environmental sciences study design

All studies must disclose on these points even when the disclosure is negative.

Study description	This study is the first whole genome population genetic analyses of A. gambiae across Africa that includes remote island populations with the goal of generating a more inclusive view of the species' evolutionary history.
Research sample	We used N=111 Anopheles gambiae specimens (N=40 from Mali, N=5 from Cameroon, N=6 from Tanzania, N=6 from Zambia, and N=54 from the Comoros) from the Vector Genetics Laboratory archive mosquito DNA collection for the study.
Sampling strategy	Samples from Mali and Cameroon were collected as female adults inside houses using mouth aspirators in August 2006. Samples from the Comoros were collected as larvae inside cisterns using scoops and transfer pipets in February 2011. Tanzania samples were collected 2012 and Zambia samples in April and May 2015 as adults by pyrethroid spray catch collection.
Data collection	Samples from Mali and Cameroon were collected as female adults inside houses using mouth aspirators in August 2006. Samples from the Comoros were collected as larvae inside cisterns using scoops and transfer pipets in February 2011. Tanzania samples were collected 2012 and Zambia samples in April and May 2015 as adults by pyrethroid spray catch collection.
Timing and spatial scale	Samples from Mali and Cameroon were collected as female adults inside houses using mouth aspirators in August 2006. Samples from the Comoros were collected as larvae inside cisterns using scoops and transfer pipets in February 2011. Tanzania samples were collected 2012 and Zambia samples in April and May 2015 as adults by pyrethroid spray catch collection.
Data exclusions	We excluded samples that appeared to be from other mosquito species based on the DIS report.
Reproducibility	No unusual attempts.
Randomization	No randomization was done, because the affiliation with the geographical groups was the essence of the study.
Blinding	No blinding was done.

Did the study involve field work? ☐ Yes ☒ No

Reporting for specific materials, systems and methods

We require information from authors about some types of materials, experimental systems and methods used in many studies. Here, indicate whether each material, system or method listed is relevant to your study. If you are not sure if a list item applies to your research, read the appropriate section before selecting a response.

Materials & experimental systems

n/a	Involved in the study	
☒	☐	Antibodies
☒	☐	Eukaryotic cell lines
☒	☐	Palaeontology
☐	☒	Animals and other organisms
☒	☐	Human research participants
☒	☐	Clinical data

Methods

n/a	Involved in the study	
☒	☐	ChIP-seq
☒	☐	Flow cytometry
☒	☐	MRI-based neuroimaging

Animals and other organisms

Policy information about studies involving animals; ARRIVE guidelines recommended for reporting animal research

Laboratory animals	Only mosquitoes from the species An. gambiae were used.
Wild animals	No animals were brought into captivity.
Field-collected samples	Mosquitoes collected in the field were sacrificed on site.
Ethics oversight	No ethical approval was required.

Note that full information on the approval of the study protocol must also be provided in the manuscript.
